# Functional Magnetic Resonance Imaging for Investigating the Role of the Hippocampus in Migraine with Aura

**DOI:** 10.3390/diagnostics16071111

**Published:** 2026-04-07

**Authors:** Mojsije Radović, Marko Daković, Aleksandra Radojičić, Igor Petrušić

**Affiliations:** 1 Faculty of Medicine, University of Belgrade, 11000 Belgrade, Serbia; dr.mojsije.radovic@gmail.com (M.R.); aleksandraradojicic@gmail.com (A.R.); 2 Laboratory for Advanced Analysis of Neuroimages, Faculty of Physical Chemistry, University of Belgrade, 11000 Belgrade, Serbia; marko@ffh.bg.ac.rs; 3 Headache Center, Neurology Clinic, University Clinical Center of Serbia, 11000 Belgrade, Serbia

**Keywords:** headache, dysphasia, aura phenomenology, visual symptoms

## Abstract

**Background/Objectives**: Migraine with aura (MwA) is a heterogeneous disorder comprising pure visual aura (MwAv) and more complex phenotypes with additional somatosensory and/or dysphasic symptoms (MwAvsd). Previous structural magnetic resonance imaging (MRI) studies have demonstrated hippocampal subfield volume reductions associated with aura complexity, suggesting a role for the hippocampus in MwA pathophysiology. However, functional network mechanisms underlying these structural differences remain unclear. This study aimed to investigate hippocampal resting-state functional connectivity (FC) in MwA subtypes and healthy controls (HCs), and to determine whether hippocampal connectivity patterns differ according to aura complexity. **Methods**: In this comparative cross-sectional study, 27 patients with MwAvsd, 18 with MwAv, and 29 age- and sex-matched HCs underwent resting-state functional MRI on a 3T scanner. Seed-based FC analyses were performed using both hippocampi as regions of interest. **Results**: MwAvsd patients demonstrated significantly increased FC between the right hippocampus and the left dorsal parietal cortex and right sensory association cortex compared with MwAv patients. In contrast, MwAv patients showed increased FC between the left hippocampus and the right dorsolateral prefrontal cortex compared with MwAvsd patients. Additionally, MwAv patients exhibited stronger FC between the left hippocampus and bilateral anterior prefrontal cortices and the left angular cortex compared with HCs. No other significant hippocampal FC differences were observed. **Conclusions**: Hippocampal FC is altered in MwA and varies according to aura phenotype. Complex aura is characterized by enhanced hippocampal coupling with multisensory integration regions and reduced connectivity with executive control areas, whereas pure visual aura demonstrates increased hippocampal–prefrontal and hippocampal–parietal associative connectivity compared with HCs. These findings suggest that the hippocampus might serve as a target for future neuromodulatory and therapeutic investigations in MwA patients.

## 1. Introduction

Migraine with aura (MwA) represents a heterogeneous clinical entity characterized by transient focal neurological symptoms that most commonly include visual disturbances, but may also involve somatosensory and higher cortical dysfunctions [1,2]. Approximately one-third of individuals with migraine experience aura, yet the clinical expression of MwA varies considerably across patients, ranging from pure visual aura (MwAv) to more complex phenotypes combining visual, somatosensory, and dysphasic symptoms (MwAvsd) [3,4]. This heterogeneity likely reflects distinct neurobiological mechanisms and network-level differences [5,6,7], underscoring the need for stratified neuroimaging approaches that account for aura complexity [8]. In addition, previous neuroimaging and electrophysiological studies have demonstrated that patients with more complex aura phenotypes exhibit structural and functional alterations in visual, somatosensory, and language-related cortical regions, suggesting that MwA subtypes should be investigated separately rather than as a single group [5,9,10,11,12,13].

Recent studies have increasingly implicated the hippocampus in migraine pathophysiology [14,15,16,17]. Although traditionally associated with episodic memory and spatial navigation, the hippocampus is now recognized as a multimodal integrative structure involved in language processing, sensory integration, and large-scale network coordination [18,19,20]. Structural neuroimaging studies have reported associations between hippocampal volume and migraine characteristics, including attack frequency, cognitive performance, and white matter abnormalities [16,21,22,23]. However, until recently, the role of the hippocampus in MwA, particularly in relation to aura complexity, remained largely unexplored [15]. In our previous structural magnetic resonance imaging (MRI) investigation, hippocampal subfield segmentation revealed that MwAvsd patients exhibited significantly smaller bilateral hippocampal volumes, particularly within subiculum and CA1-related regions, compared with MwAv patients and healthy controls (HCs) [15]. Moreover, hippocampal volumes negatively correlated with aura complexity, suggesting that hippocampal structural alterations may contribute to the emergence of non-visual aura symptoms and to the broader network dysfunction underlying complex aura phenotypes [15,24]. However, structural imaging alone cannot elucidate the functional network mechanisms through which the hippocampus may influence aura complexity. Moreover, although neuroimaging studies have been conducted previously to investigate MwA, questions remained whether patients with more complex aura demonstrate altered functional connectivity (FC) between the hippocampus and cortical regions, particularly in the somatosensory and language networks. Functional MRI, particularly seed-based connectivity analysis, provides a powerful method to investigate these network-level interactions and to determine whether hippocampal connectivity patterns differ between MwA subtypes and healthy individuals [25,26,27].

The present study represents the next phase of our investigation into hippocampal involvement in MwA. Using resting-state seed-based functional MRI, we examined FC between the left and right hippocampus and the rest of the brain in MwAv, MwAvsd and HCs. The primary objective was to determine whether hippocampal connectivity patterns differ between these groups. We hypothesized that patients with more complex aura phenotypes would demonstrate altered FC between the hippocampus and cortical regions involved in somatosensory and language processing, reflecting the hippocampus’s role as a multimodal integrative hub in the MwA pathophysiology.

## 2. Materials and Methods

### 2.1. Participants

This is a comparative study between patients who have episodic MwA (IHCD-3, code 1.2.1) [1] and HCs using resting-state functional MRI data. MwA patients and HCs included in the study were drawn from the cohort enrolled in a previous structural neuroimaging study [15]. Patients with MwA were recruited from among those who consecutively visited the Headache Center at the University Clinical Centre of Serbia. The sample size was based on the available data and previous literature [6,15]. We performed a sample size calculation (*N* = z^2^ × (1 − *p*)/d^2^) based on a confidence level of 95% (z = 1.96), a margin of error (d) of 5%, and an estimated MwAvsd population proportion (*p*) of 1.5%, which showed that 23 or more participants were required for a confidence level of 95% for the measured values in the MwAvsd population [15]. The following inclusion criteria were applied: (1) 18–55 years of age, (2) suffering from episodic MwA for more than two years before enrolment in the study, (3) absence of migraine preventive therapy, and (4) patient consent to participate in the study. A trained headache specialist examined and interviewed all MwA patients who agreed to participate in the study. The following exclusion criteria were applied: (1) the presence of other types of MwA (such as migraine with brainstem aura, hemiplegic or retinal migraine), (2) the presence of any other neurological (except occasional migraine without aura and tension-type headaches), cardiovascular or endocrine diseases, (3) reported claustrophobia or inability to perform MRI examination, and (4) structural abnormalities on MRI scan. MwA patients were subdivided into MwAv and MwAvsd subgroups for more profound analysis. HCs were voluntarily recruited from hospital and university staff, as well as their friends and relatives, and balanced with MwA patients in terms of sex and age. All HCs underwent interviews and physical exams to check their health status and detect any exclusion criteria. Furthermore, HCs had no family members who suffered from a migraine.

The study was approved by the Scientific Ethics Committee of the Clinical Center and Neurology Clinic (reference number: 23-690). Eligible patients signed an informed consent to participate in the study.

### 2.2. MRI Data Acquisition and Post-Processing

The MRI examination was performed on a 3 T Scanner (MAGNETOM Skyra, Siemens, Erlangen, Germany). Earplugs and foam padding were used to minimize scanner noise and restrict head motion. When the resting-state functional MRI acquisition started, individuals were asked to stay awake, look at the black cross on the white screen in front of them and think of nothing. Protocol for MRI examination was: (1) 3D T1 (repetition time (TR) = 2300 ms, echo time (TE) = 2.98 ms, flip angle = 9°, 130 slices with voxel size 1 × 1 × 1 mm^3^, acquisition matrix 512 × 512, FOV = 256 × 256 mm^2^, scan time = 5:12 min), (2) T2 weighted spin echo [T2W] in an axial plane (TR = 4800 ms, TE = 92 ms, flip angle (FA) = 90°, acquisition matrix 384 × 265, FOV = 256 × 256 mm^2^, slice thickness = 5 mm), and (3) T2*-weighted echo-planar (EPI) functional imaging, sensitive to the blood-oxygen-level-dependent (BOLD) signal (TR = 2300 ms, TE = 35 ms, flip angle = 90°, voxel size 3 × 3 × 3 mm^3^, 36 slices, number of functional volumes = 280, scan time = 10 min). BOLD images were acquired in descending order. The MRIs were inspected visually for artifacts and blurring. In the preparative process, T1 images were subjected to brain extraction using the FSL BET routine with an option of robust brain center estimation. The same routine was performed on the magnitude field map images, now with default options. FSL fsl_prepare_fieldmap was used to convert field map phase images in rad/s units. Motion correction and high-pass temporal filtering were conducted in FSL FEAT1 (http://www.fmrib.ox.ac.uk/fsl, accessed on 9 October 2025). Functional image processing included correction for slice-timing and head motion using rigid-body transformations with 6 degrees of freedom using FSL MCFLIRT2. By inspection of the motion parameters after completion of the procedure (in FSL MCFLIRT), we established that no subject showed relative movement larger than half of the voxel dimensions (3 × 3 × 3 mm^3^). As the reference for slice timing correction, we used the first volume in the set. The patient’s functional images were co-registered to the structural images in native space and then normalized to the MNI152 T1 template (Montreal Neurological Institute, Montreal, QC, Canada). Then, binary masks of the left and right hippocampi were selected as seed regions using FSL Eyes and the fslmaths script. The Harvard-Oxford Subcortical Structural Atlas was used to define both hippocampi. Seed-based analyses were performed. Mean time courses from all voxels within the unilateral hippocampus were extracted and used as reference time courses. FLAME (FMRIB’s Local Analysis of Mixed Effects), as the higher-level modeling tool, was used for group-level functional MRI analysis. It is designed to model multi-level hierarchies, such as subjects within groups, to make inferences about group-level effects (Z-statistics) [28].

T2W images were used only to exclude the presence of brain lesions or any structural abnormalities. MwA patients did not experience a migraine 72 h before and 72 h after the MRI scan.

### 2.3. Statistical Analyses

Demographic and clinical data were compared between MwAv, MwAvsd and HCs using the independent-sample *t*-test for continuous parametric data, Mann–Whitney U for continuous non-parametric data and chi-square test for categorical data, as appropriate. The Shapiro–Wilk test was used to test whether a data set is normally distributed. All analyses were performed using SPSS software (version 21.0).

The Pearson correlation coefficient was calculated between the mean time courses of each reference and voxel. Subsequently, Fisher’s z-transformation was applied to normalize the original correlation maps. The FC maps for the two regions were established, and the difference between the group-level functional maps was then analyzed [28]. Brain regions were considered significant within a cluster-wise threshold of *p* < 0.05 after the family-wise error was used for correction of multiple comparisons and a cluster size of >50.

To investigate the association between FC in regions differing between the MwAv and MwAvsd groups and migraine-related variables (attack frequency, disease duration, and headache intensity), as well as the volumes of the left and right hippocampi, Pearson correlation analysis was performed to assess the relationships between Z-scores and both clinical parameters and whole hippocampal volumes.

## 3. Results

### 3.1. Study Population

A total of 27 MwAvsd and 18 MwAv patients, as well as 29 HCs, were studied. They were balanced in age (35.4 ± 9.2 vs. 38.2 ± 8.4 vs. 36.6 ± 8.6 years, *p* = 0.599) and sex (70.4% vs. 66.7% vs. 69.0% of females, *p* = 0.966). Clinical characteristics of MwAvsd and MwAv subgroups are shown in Table 1.

### 3.2. Seed-Based FC Results

**Figure 1:**
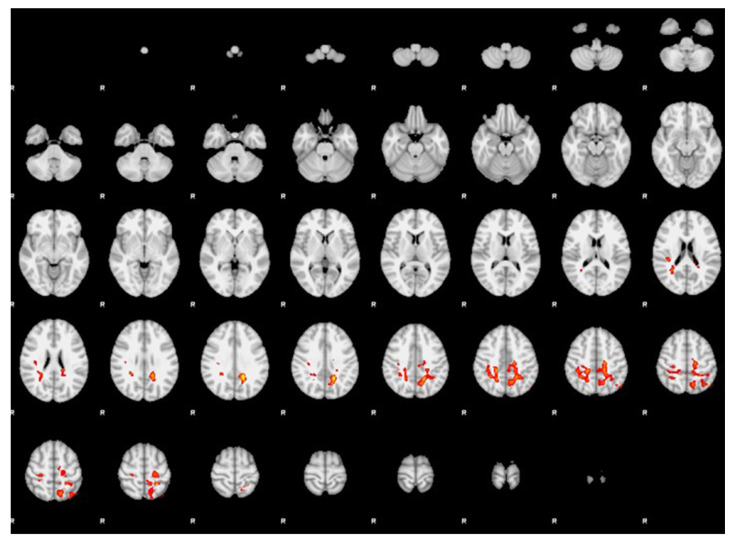
Functional connectivity analysis results using the right hippocampus as the seed region in participants with MwAvsd compared with MwAv (cluster significance threshold *p* < 0.05, family-wise error-corrected). Participants with MwAvsd showed significantly stronger functional connectivity than those with MwAv between the right hippocampus and the left dorsal parietal cortex, as well as the right sensory association cortex.

Participants with MwAvsd showed significantly stronger FC compared to MwAv between the right hippocampus and the left dorsal parietal cortex and right sensory association cortex (Table 2, Figure 1).

**Figure 2:**
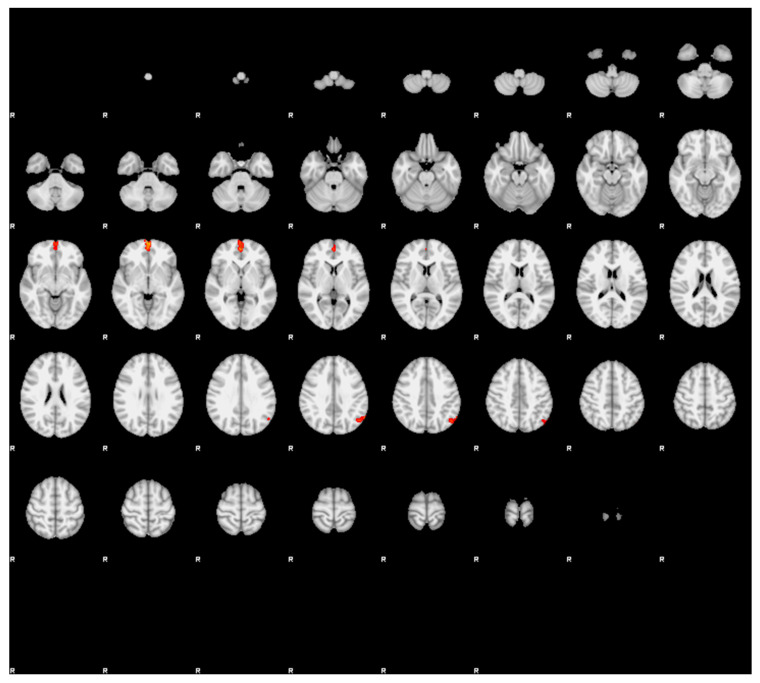
Functional connectivity analysis results using the left hippocampus as the seed region in participants with MwAvsd compared with MwAv (cluster significance threshold *p* < 0.05, family-wise error-corrected). Participants with MwAv showed significantly stronger functional connectivity than those with MwAvsd between the left hippocampus and the right dorsolateral prefrontal cortex.

**Figure 3:**
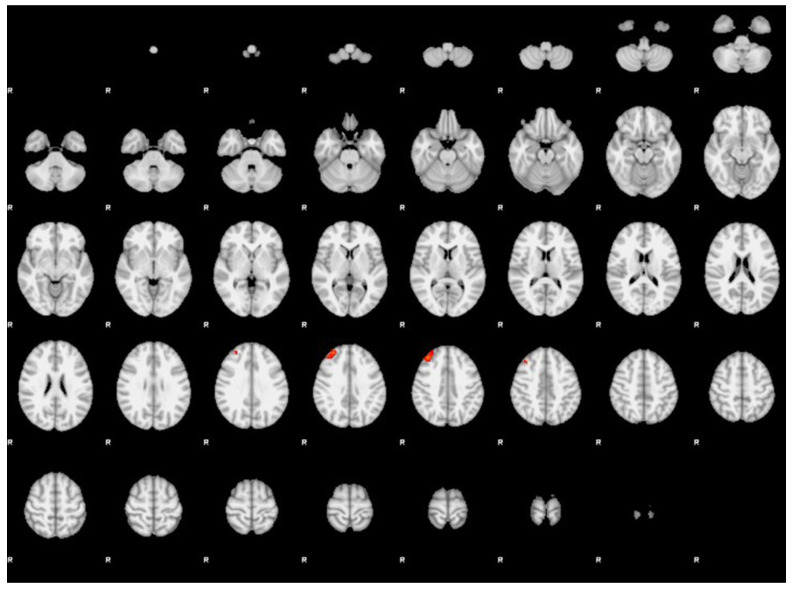
Functional connectivity analysis results using the left hippocampus as the seed region in participants with MwAv compared with HCs (cluster significance threshold *p* < 0.05, family-wise error-corrected). Participants with MwAv showed significantly stronger functional connectivity than HCs between the left hippocampus and bilateral anterior prefrontal cortices and left angular cortex.

Participants with MwAv showed significantly stronger FC compared to MwAvsd between the left hippocampus and the right dorsolateral prefrontal cortex (Table 2, Figure 2). In addition, participants with MwAv showed significantly stronger FC compared to HCs between the left hippocampus and bilateral anterior prefrontal cortices and left angular cortex (Table 2, Figure 3). There were no other significant FCs between the right or left hippocampus and other brain regions when comparing MwAv group to MwAvsd or HCs.

There was no significant association of attack frequency, disease duration, headache intensity, volumes of the whole left and right hippocampi with increased FC between the right hippocampus and left dorsal parietal cortex, the right hippocampus and right sensory association cortex, and the left hippocampus and right dorsolateral prefrontal cortex (Table 3).

## 4. Discussion

Our previous structural MRI study demonstrated that hippocampal subfield volumes are reduced in patients with more complex aura phenotypes and that these reductions correlate with aura complexity [15]. The present seed-based resting-state functional results extend these observations by showing that hippocampal FC is also altered in a phenotype-dependent manner. Therefore, the convergence of structural and functional findings within the same individuals suggests that hippocampal involvement in MwA is not limited to local morphometric changes but also reflects broader network-level reorganization, supporting an important role of the hippocampus in MwA pathophysiology at the network level.

Previous neuroimaging studies have reported altered hippocampal FC in migraine without aura, including changes in FCs with the prefrontal cortex, posterior insula, supplementary motor area, inferior parietal lobe, and visual cortex [29,30]. However, none of the studies have focused specifically on MwA or on differences between aura phenotypes. The present findings demonstrate that hippocampal FC is not only altered in MwA but also varies with aura complexity. Therefore, this study may pave the way towards multimodal neuroimaging investigations of the hippocampal role in well-characterized MwA patients, further elucidating its role in MwA pathophysiology.

The hippocampus is anatomically and functionally connected to the visual association cortex, temporoparietal regions, thalamus, and limbic structures, positioning it as a hub that modulates distributed cortical activity [18,19]. In the context of MwA, such a role is particularly relevant, given that aura involves transient disturbances across multiple sensory and cognitive domains [31]. From a mechanistic perspective, altered hippocampal connectivity may influence the susceptibility and propagation of cortical spreading depolarization, the electrophysiological event underlying aura [32]. The hippocampus has extensive reciprocal connections with association cortices and subcortical structures that modulate cortical excitability [33]. Furthermore, the hippocampus is one of the most epileptogenic areas in the brain. Therefore, we could hypothesize that changes in hippocampal network interactions could contribute to differences in how aura symptoms emerge and spread across cortical regions [33,34]. In patients with MwAvsd, more pronounced hippocampal connectivity alterations may reflect a broader network vulnerability that facilitates involvement of somatosensory and language networks in addition to the visual cortex.

MwAvsd demonstrated increased FC between the right hippocampus and the left dorsal parietal cortex as well as the right sensory association cortex, compared with patients with pure visual aura. The dorsal parietal cortex, particularly within the superior parietal lobule and intraparietal regions, is central to multisensory integration, spatial attention, and sensorimotor transformation [35]. It plays an important role in integrating visual and somatosensory inputs and in coordinating distributed cortical networks involved in perception and action [36,37]. Enhanced coupling between the right hippocampus and dorsal parietal regions in MwAvsd patients may reflect increased interaction between memory-contextual processing systems and multisensory integration networks. Therefore, it could be hypothesized that such strengthened connectivity could facilitate the propagation of aberrant activity across visual and somatosensory domains during aura, potentially contributing to the emergence of non-visual symptoms. From a pathophysiological standpoint, this pattern is consistent with the notion that complex aura involves broader network recruitment beyond the primary visual cortex [31]. Similarly, increased functional coupling between the right hippocampus and sensory association cortex may indicate enhanced synchronization between limbic-contextual systems and multimodal sensory processing networks [38,39]. This could reflect a compensatory mechanism aimed at maintaining sensory coherence in the presence of network instability, or alternatively, a maladaptive reorganization that predisposes to the spread of cortical spreading depolarization into non-visual cortical territories. In contrast, MwAvsd patients showed decreased connectivity between the left hippocampus and the right dorsolateral prefrontal cortex compared with MwAv patients. The dorsolateral prefrontal cortex is a key node in executive control and top-down modulation of sensory and limbic systems [40]. Reduced hippocampal–dorsolateral prefrontal cortex FC may therefore reflect diminished top-down regulation of limbic and sensory networks in patients with more complex aura. This decreased coupling might impair the brain’s ability to constrain or modulate aberrant cortical activity, potentially allowing aura phenomena to involve additional cortical systems such as somatosensory and language networks. Moreover, the dorsolateral prefrontal cortex has been implicated in pain modulation and migraine chronification [41,42,43]. Therefore, reduced connectivity with the hippocampus may thus contribute not only to aura complexity but also to broader cognitive and affective features of migraine. Taken together, these findings suggest that complex aura is associated with a shift toward stronger hippocampal interactions with multisensory integration regions and weaker coupling with executive control networks. This imbalance may create a neural environment that facilitates the spread and diversification of aura symptoms across multiple functional domains.

Compared with HCs, MwAv patients demonstrated increased FC between the left hippocampus and bilateral anterior prefrontal cortices, as well as the left angular cortex. These findings indicate that even in the absence of somatosensory or language aura symptoms, hippocampal network organization is altered in MwAv. Moreover, the anterior prefrontal cortex is involved in higher-order cognitive processes, including prospective memory, complex decision-making, and integration of internal and external information over extended timescales [44,45]. Therefore, increased hippocampal–anterior prefrontal FC in MwAv patients may reflect enhanced interaction between memory-contextual systems and high-level cognitive control networks. In addition, the angular gyrus, located within the inferior parietal lobule, is a multimodal association area involved in language processing, semantic integration, attention, and aspects of visuospatial cognition [46]. It also participates in the default mode and frontoparietal networks and serves as an interface between sensory and cognitive systems [47]. Increased FC between the left hippocampus and the left angular cortex in MwAv patients suggests altered coupling between memory-related and associative cortical regions. Although MwAv patients do not experience dysphasic aura by definition, the involvement of the angular gyrus in language and semantic processing raises the possibility that subtle network changes extend beyond strictly visual systems. Moreover, increased hippocampal-prefrontal and hippocampal-angular FC in pure visual aura may reflect compensatory or early-stage network adaptations that help limit aura to visual brain domains.

These findings align with emerging models of MwA that emphasize large-scale network dysfunction rather than isolated regional abnormalities [6]. The hippocampus, through its extensive reciprocal connections with cortical and subcortical regions, is well positioned to influence the initiation of aura phenomena and modulate cortical spreading depolarization followed by depression. The present results, therefore, suggest that hippocampal structural and functional alterations are closely linked to the heterogeneity of MwA and may represent a key substrate underlying differences in aura expression. Moreover, it supports the concept that MwA is not a uniform disorder but comprises biologically distinct subtypes. Therefore, patients with pure visual aura and those with additional somatosensory and dysphasic symptoms appear to differ not only clinically but also in the organization of hippocampal networks.

Key strength of this study include a well-defined MwA cohort. Additionally, participants did not have comorbidities and were not receiving preventive migraine therapy, reducing potential confounding effects. Furthermore, adding functional MRI analyses upon previous structural findings in the same patient cohort is a major strength of the present work. This design minimizes inter-cohort variability and allows direct interpretation of functional findings in the context of previously documented structural differences. Such an approach is essential for advancing mechanistic understanding of MwA heterogeneity and for identifying network-level biomarkers that may distinguish clinically meaningful subtypes [6]. Nonetheless, several limitations must be acknowledged. First, although the use of a single well-characterized cohort is a strength, replication in independent cohorts will be necessary to confirm the observed connectivity patterns. Second, the cross-sectional design precludes causal inference regarding the relationship between structural and functional alterations. Longitudinal studies could clarify whether hippocampal changes precede the development of complex aura or arise as a consequence of repeated attacks. Third, resting-state fMRI provides indirect measures of FC and cannot determine the directionality of interactions between regions. Combining resting-state analyses with task-based paradigms or effective connectivity methods may provide additional insights into hippocampal network dynamics. In addition, complementary analyses, such as independent component analysis, graph-theoretical metrics, or effective connectivity approaches, could further contribute to understanding the role of the hippocampus in MwA pathophysiology. Moreover, investigating hippocampal FC during different phases of the migraine cycle may also provide insight into state-dependent versus trait-like alterations. In addition, potential confounding effects of sex and handedness should be considered when interpreting the present findings. Although the study groups were balanced for sex, thereby reducing the likelihood of a systematic gender bias, sex-related differences in brain network organization and migraine pathophysiology are well documented and may still contribute to inter-individual variability in FC patterns. Additionally, handedness was not systematically assessed in this cohort, representing a limitation of the study. Hemispheric lateralization of cognitive and sensory functions, as well as hippocampal network organization, may differ between right- and left-handed individuals. Given that the present results include lateralized connectivity patterns, variability related to handedness cannot be excluded. Future studies incorporating detailed assessment of handedness, along with larger sample sizes, will be important to clarify its potential role in shaping hippocampal FC in MwA.

## 5. Conclusions

Hippocampal FC is altered in MwA and differs according to aura phenotype, supporting its role in the network-level mechanisms underlying aura heterogeneity. These findings highlight the hippocampus as a potential biomarker and target for future mechanistic and translational studies.

## Figures and Tables

**Table 1:** Characteristics of MwAvsd and MwAv subgroups.

Variables	MwAvsd (*n* = 27)	MwAv (*n* = 18)	*p*-Value
Disease duration in years, mean ± sd	18.2 ± 10.9	16.3 ± 9.9	0.538
MwA frequency per year, mean ± sd	8.3 ± 9.1	3.9 ± 2.5	0.244
Photophobia, number of patients (%)	26 (96)	15 (83)	0.286
Phonophobia, number of patients (%)	16 (59)	6 (33)	0.130
Nausea, number of patients (%)	17 (63)	8 (44)	0.241
Headache intensity, mean ± sd	6.7 ± 2.2	6.4 ± 2.5	0.712
MwoA, number of patients (%)	14 (52)	6 (33)	0.359

MwAvsd—migraine with visual and somatosensory and/or dysphasic auras, MwAv—migraine with pure visual aura, MwA—migraine with aura, MwoA—migraine without aura, sd—standard deviation. Statistics—independent-sample *t*-test for continuous parametric data, Mann–Whitney U for continuous non-parametric data, and chi-square test for categorical data.

**Table 2:** Abnormal FC of the right or left hippocampus in MwA subgroups.

Cluster Voxels	*p* Value	Z-Max	Peak MNI Coordinates			Brain Region
			X	Y	Z	
FC of the right hippocampus in MwAvsd > MwAv						
2227	<0.001	4.05	−18	−50	34	Left dorsal parietal cortex
1055	0.032	3.62	20	−36	46	Right sensory association cortex
FC of the left hippocampus in MwAv > MwAvsd						
212	0.031	3.46	36	38	40	Right dorsolateral prefrontal cortex
FC of the left hippocampus in MwAv > HCs						
321	0.002	3.84	0	62	−4	Bilateral anterior prefrontal cortices
206	0.037	3.56	−60	−60	38	Left angular cortex

Z-max—the highest Z-score value detected within a specific cluster, MNI—Montreal Neurological Institute, Peak MNI—the coordinate of the location of the peak of the voxel cluster, FC—functional connectivity, MwAvsd—migraine with visual and somatosensory and/or dysphasic auras, MwAv—migraine with pure visual aura, HCs—healthy controls.

**Table 3:** Relationships between significant FCs in MwA patients and both clinical parameters and whole hippocampal volumes.

	Left Dorsal Parietal Cortex	Right Sensory Association Cortex	Right Dorsolateral Prefrontal Cortex
MwA attack frequency per year	rho = −0.112, *p* = 0.476	rho = −0.120, *p* = 0.443	rho = −0.009, *p* = 0.955
Disease duration in years	rho = −0.125, *p* = 0.424	rho = −0.251, *p* = 0.105	rho = −0.043, *p* = 0.784
Headache intensity	rho = −0.164, *p* = 0.293	rho = 0.069, *p* = 0.658	rho = −0.244, *p* = 0.115
Volume of the whole left hippocampus	rho = 0.018, *p* = 0.909	rho = 0.127, *p* = 0.418	rho = 0.108, *p* = 0.490
Volume of the whole right hippocampus	rho = 0.001, *p* = 0.994	rho = −0.165, *p* = 0.291	rho = 0.090, *p* = 0.566

FC—functional connectivity, MwA—migraine with aura.

## Data Availability

The datasets presented in this article are not readily available because the data are part of an ongoing study. Requests to access the datasets should be directed to the corresponding author.

## Abbreviations

The following abbreviations are used in this manuscript:

FC	functional connectivity
HCs	healthy controls
MRI	magnetic resonance imaging
MwA	Migraine with aura
MwAv	MwA patients with pure visual aura
MwAvsd	MwA patients with visual, somatosensory, and dysphasic aura

## References

[1] Headache Classification Committee of the International Headache Society (IHS) (2018). The International classification of Headache disorders. Cephalalgia.

[2] Goadsby P.J., Holland P.R., Martins-Oliveira M., Hoffmann J., Schankin C., Akerman S. (2017). Pathophysiology of Migraine: A Disorder of Sensory Processing. Physiol. Rev..

[3] Petrusic I., Viana M., Dakovic M., Goadsby P.J., Zidverc-Trajkovic J. (2018). Proposal for a migraine aura complexity score. Cephalalgia.

[4] Viana M., Sances G., Linde M., Ghiotto N., Guaschino E., Allena M., Terrazzino S., Nappi G., Goadsby P.J., Tassorelli C. (2017). Clinical features of migraine aura: Results from a prospective diary-aided study. Cephalalgia.

[5] Coppola G., Renzo A.D., Tinelli E., Petolicchio B., Parisi V., Serrao M., Porcaro C., Fiorelli M., Caramia F., Schoenen J. (2021). Thalamo-cortical networks in subtypes of migraine with aura patients. J. Headache Pain.

[6] Mitrovic K., Petrusic I., Dakovic M., Savić A. (2023). Migraine with aura detection and subtype classification using machine learning algorithms and morphometric magnetic resonance imaging data. Front. Neurol..

[7] Messina R., Rocca M.A., Goadsby P.J., Filippi M. (2023). Insights into migraine attacks from neuroimaging. Lancet Neurol..

[8] Petrusic I., Viana M., Dakovic M., Zidverc-Trajkovic J. (2019). Application of the Migraine Aura Complexity score (MACS): Clinical and Neuroimaging Study. Front. Neurol..

[9] Petrusic I., Jovanovic V., Kovic V., Savic A.M. (2022). P3 latency as a biomarker for the complexity of migraine with aura: Event-related potential study. Cephalalgia.

[10] Coppola G., Bracaglia M., Di Lenola S., Di Lorenzo C., Serrao M., Parisi V., Di Renzo A., Martelli F., Fadda A., Schoenen J. (2015). Visual evoked potentials in subgroups of migraine with aura patients. J. Headache Pain.

[11] Silvestro M., Tessitore A., Di Nardo F., Scotto di Clemente F., Trojsi F., Cirillo M., Esposito F., Tedeschi G., Russo A. (2022). Functional connectivity changes in complex migraine aura: Beyond the visual network. Eur. J. Neurol..

[12] Abagnale C., Di Renzo A., Sebastianelli G., Casillo F., Tinelli E., Giuliani G., Tullo M.G., Serrao M., Parisi V., Fiorelli M. (2023). Whole brain surface-based morphometry and tract-based spatial statistics in migraine with aura patients: Difference between pure visual and complex auras. Front. Hum. Neurosci..

[13] Petrušić I., Savić A., Mitrović K., Bačanin N., Sebastianelli G., Secci D., Coppola G. (2024). Machine learning classification meets migraine: Recommendations for study evaluation. J. Headache Pain.

[14] Cankaya S., Ayyildiz B., Sayman D., Duran U., Ucak D., Karaca R., Ayyildiz S., Oktem E.O., Lakadamyalı H., Sayman C. (2025). Hippocampal connectivity dynamics and volumetric alterations predict cognitive status in migraine: A resting-state fMRI study. Neuroimage.

[15] Petrušić I., Radović M., Daković M., Radojičić A., Coppola G. (2024). Subsegmentation of the hippocampus in subgroups of migraine with aura patients: Advanced structural neuroimaging study. J. Headache Pain.

[16] He M., Kis-Jakab G., Komaromy H., Perlaki G., Orsi G., Bosnyák E., Rozgonyi R., John F., Trauninger A., Eklics K. (2023). The volume of the thalamus and hippocampus in a right-handed female episodic migraine group. Front. Neurol..

[17] Feng Q., Chen W., Ke J., Geng D., Xiong X., Dai L., Zhao H., Hu C. (2025). Volumetric analysis of hippocampal subregions in migraine without aura: An exploratory study on mechanisms underlying migraine chronification. J. Headache Pain.

[18] Dalton M.A., D’Souza A., Lv J., Calamante F. (2022). New insights into anatomical connectivity along the anterior-posterior axis of the human hippocampus using in vivo quantitative fibre tracking. eLife.

[19] Chan R.W., Leong A.T.L., Ho L.C., Gao P.P., Wong E.C., Dong C.M., Wang X., He J., Chan Y.-S., Lim L.W. (2017). Low-frequency hippocampal-cortical activity drives brain-wide resting-state functional MRI connectivity. Proc. Natl. Acad. Sci. USA.

[20] Piai V., Anderson K.L., Lin J.J., Dewar C., Parvizi J., Dronkers N.F., Knight R.T. (2016). Direct brain recordings reveal hippocampal rhythm underpinnings of language processing. Proc. Natl. Acad. Sci. USA.

[21] Maleki N., Becerra L., Brawn J., McEwen B., Burstein R., Borsook D. (2013). Common hippocampal structural and functional changes in migraine. Brain Struct. Funct..

[22] Chong C.D., Dumkrieger G., Schwedt T.J. (2017). Structural co-variance patterns in migraine: A cross-sectional study exploring the role of the Hippocampus. Headache.

[23] Tsai C.L., Chou K.H., Lee P.L., Liang C.-S., Kuo C.-Y., Lin G.-Y., Lin Y.-K., Hsu Y.-C., Ko C.-A., Yang F.-C. (2023). Shared alterations in hippocampal structural covariance in subjective cognitive decline and migraine. Front. Aging Neurosci..

[24] Mitrović K., Savić A., Radojičić A., Daković M., Petrušić I. (2023). Machine learning approach for Migraine Aura Complexity Score prediction based on magnetic resonance imaging data. J. Headache Pain.

[25] Smitha K.A., Akhil Raja K., Arun K.M., Rajesh P.G., Thomas B., Kapilamoorthy T.R., Kesavadas C. (2017). Resting state fMRI: A review on methods in resting state connectivity analysis and resting state networks. Neuroradiol. J..

[26] Colombo B., Rocca M.A., Messina R., Guerrieri S., Filippi M. (2015). Resting-state fMRI functional connectivity: A new perspective to evaluate pain modulation in migraine?. Neurol. Sci..

[27] Skorobogatykh K., van Hoogstraten W.S., Degan D., Prischepa A., Savitskaya A., Ileen B.M., Bentivegna E., Skiba I., D’Acunto L., Ferri L. (2019). Functional connectivity studies in migraine: What have we learned?. J. Headache Pain.

[28] Worsley K.J., Jezzard P., Matthews P.M., Smith S.M. (2001). Statistical analysis of activation images. Book Functional MRI: An Introduction to Methods.

[29] Wei H.L., Chen J., Chen Y.C., Yu Y.-S., Zhou G.-P., Qu L.-J., Yin X., Li J., Zhang H. (2020). Impaired functional connectivity of limbic system in migraine without aura. Brain Imaging Behav..

[30] Zhu Y., Dai L., Zhao H., Ji B., Yu Y., Dai H., Hu C., Wang X., Ke J. (2021). Alterations in Effective Connectivity of the Hippocampus in Migraine without Aura. J. Pain Res..

[31] Karsan N., Silva E., Goadsby P.J. (2023). Evaluating migraine with typical aura with neuroimaging. Front. Hum. Neurosci..

[32] Ayata C., Lauritzen M. (2015). Spreading Depression, Spreading Depolarizations, and the Cerebral Vasculature. Physiol. Rev..

[33] Arren Hill A., Amendolara A.B., Small C., Guzman S.C., Pfister D., McFarland K., Settelmayer M., Baker S., Donnelly S., Payne A. (2024). Metabolic Pathophysiology of Cortical Spreading Depression: A Review. Brain Sci..

[34] Florez C., Lukankin V., Sugumar S., McGinn R., Zhang Z., Zhang L., Carlen P. (2015). Hypoglycemia-induced alterations in hippocampal intrinsic rhythms: Decreased inhibition, increased excitation, seizures and spreading depression. Neurobiol. Dis..

[35] Sereno M.I., Huang R.-S. (2014). Multisensory maps in parietal cortex. Curr. Opin. Neurobiol..

[36] Freud E., Behrmann M., Snow J.C. (2020). What Does Dorsal Cortex Contribute to Perception?. Open Mind.

[37] Orban G.A., Sepe A., Bonini L. (2021). Parietal maps of visual signals for bodily action planning. Brain Struct. Funct..

[38] Wiener S.I., Berthoz A., Zugaro M.B. (2002). Multisensory processing in the elaboration of place and head direction responses by limbic system neurons. Brain Res. Cogn. Brain Res..

[39] Zhou L., Xu T., Feng T. (2025). The hippocampus-IPL connectivity links to ADHD traits through sensory processing sensitivity. Cereb. Cortex.

[40] Friedman N.P., Robbins T.W. (2021). The role of prefrontal cortex in cognitive control and executive function. Neuropsychopharmacology.

[41] Seminowicz D.A., Moayedi M. (2018). The dorsolateral prefrontal cortex in acute and chronic pain. J. Pain.

[42] Clemente L., Paparella G., Scannicchio S., Abbatantuono C., Tancredi G., Ladisa E., Delussi M.D., Ammendola E., Prudenzano A.M.P., de Tommaso M. (2025). Dorso lateral prefrontal cortex stimulation with TMS in chronic migraine individuals refractory to anti-CGRP monoclonal antibodies: Clinical, neuropsychological and neurophysiological effects. Cephalalgia.

[43] Mungoven T.J., Marciszewski K.K., Macefiel V.G., Macey P.M., Henderson L.A., Meylakh N. (2022). Alterations in pain processing circuitries in episodic migraine. J. Headache Pain.

[44] Preston A.R., Eichenbaum H. (2013). Interplay of hippocampus and prefrontal cortex in memory. Curr. Biol..

[45] Nobre A.C., Gresch D. (2025). How the brain shifts between external and internal attention. Neuron.

[46] Kuhnke P., Chapman C.A., Cheung V.K.M., Turker S., Graessner A., Martin S., Williams K.A., Hartwigsen G. (2022). The role of the angular gyrus in semantic cognition: A synthesis of five functional neuroimaging studies. Brain Struct. Funct..

[47] Kim H., Wang K., Cutting L.E., Willcutt E.G., Petrill S.A., Leopold D.R., Reineberg A.E., Thompson L.A., Banich M.T. (2022). The Angular Gyrus as a Hub for Modulation of Language-related Cortex by Distinct Prefrontal Executive Control Regions. J. Cogn. Neurosci..

